# Effect of IV Iloprost on Distal Flow in Buerger’s Disease: Correlation with CT Perfusion

**DOI:** 10.3390/jcm15041391

**Published:** 2026-02-10

**Authors:** Nilgün Yazıksız, Edanur Karapınar, Celal Caner Ercan, Birol Akdoğan, Gozde Oztan, Nilgün Bozbuğa

**Affiliations:** 1 Cardiovascular Surgery, Burdur Government Hospital, Burdur 15020, Turkey; 2 Radiology, Istanbul Medical Faculty, Istanbul 34093, Turkey; edanurkarapinarr@gmail.com (E.K.); cce@istanbul.edu.tr (C.C.E.); 3 Cardiovascular Surgery, Istanbul Medical Faculty, Istanbul 34093, Turkey; drbiroakdogan@gmail.com (B.A.); nilgun.bozbuga@istanbul.edu.tr (N.B.); 4 Medical Biology, Istanbul Medical Faculty, Istanbul 34093, Turkey; gozdeoztan@istanbul.edu.tr

**Keywords:** Buerger’s disease, thromboangiitisobliterans, perfusion computed tomography, intravenous iloprost

## Abstract

**Objectives**: Revascularization in thromboangiitis obliterans (TAO) is limited by distal small-vessel involvement and poor blood flow; no curative treatment exists. This study aimed to evaluate the effect of intravenous iloprost (IVI) on distal perfusion using computed tomography (CT) perfusion imaging and to correlate perfusion changes with clinical outcomes, with a focus on treatment duration. **Methods**: This retrospective cohort study was conducted at a single tertiary cardiovascular surgery center. Thirty-three patients (32 men and 1 woman) with confirmed TAO treated with IVI were screened. Clinical data, including ankle–brachial index (ABI), claudication distance, and pre- and post-treatment CT perfusion parameters, were obtained from outpatient records. Patients were grouped according to IVI duration: 0 days (n = 8), 7 days (n = 7), 14 days (n = 10), and 21 days (n = 8). One patient was excluded due to incomplete data, leaving 32 patients for analysis. **Results**: IV iloprost therapy resulted in significant improvements in ABI, claudication distance, and CT perfusion parameters, particularly in the 14- and 21-day treatment groups. No statistically significant differences were observed between the 14- and 21-day regimens; however, both were superior to shorter or no treatment. The 21-day group demonstrated the most consistent overall improvement. Treatment efficacy was independent of active smoking status, and patients with baseline ABI > 0.8 showed a more favorable response. **Conclusions**: Intravenous iloprost is clinically effective in TAO patients. Improvements in CT perfusion strongly correlate with ABI and claudication distance, suggesting that CT perfusion may serve as an early marker of treatment response and a useful adjunctive tool in TAO assessment.

## 1. Introduction

Thromboangiitis obliterans (TAO) is a non-atherosclerotic, segmental inflammatory disease that primarily affects small- and medium-sized vessels, most commonly in young male smokers [1]. Adjacent veins and peripheral nerves may also be involved, with distal ischemia and migratory thrombophlebitis representing characteristic clinical features [1]. The prevalence of TAO shows marked geographic variation, with higher rates reported in Asia and among Ashkenazi Jewish populations [2]. Although the disease has traditionally been considered male-predominant, its incidence among women has increased in parallel with rising tobacco use. Proposed contributing factors include autoimmune mechanisms, nutritional deficiencies, and genetic susceptibility [3]. Histopathologically, TAO is characterized by inflammatory thrombus formation with relative preservation of the vessel wall architecture, and immune-mediated processes, endothelial dysfunction, hypercoagulability, and infectious factors have all been implicated in disease development [3,4].

The etiopathogenesis of TAO remains incompletely understood and is widely regarded as multifactorial. The pathological process typically begins as periarteritis or periphlebitis and may progress to panarteritis and panphlebitis, accompanied by endothelial proliferation and inflammatory thrombus formation [5]. Accumulating evidence suggests that immunological mechanisms play a significant role, including increased immune complex formation, the presence of anti-endothelial antibodies, and disturbances in both cellular and humoral immunity [6,7,8,9]. In addition, endothelial dysfunction, increased expression of adhesion molecules, and elevated levels of proinflammatory cytokines appear to contribute to the persistence of vascular inflammation [6,9]. Genetic predisposition to thrombophilia, abnormalities in coagulation pathways, and potential infectious triggers further support the multifactorial nature of TAO [10].

**Figure 1:**
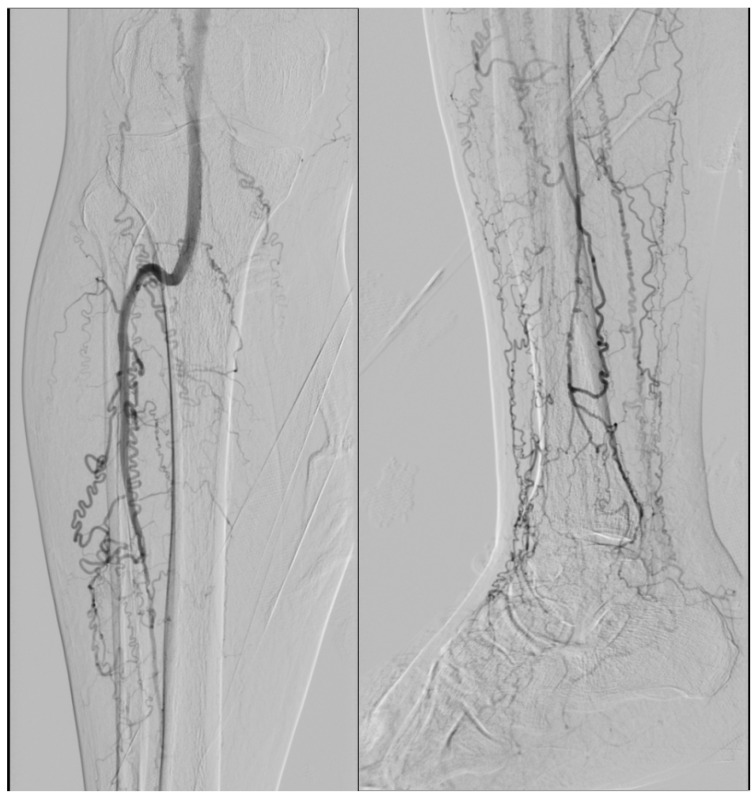
Corkscrew collaterals (from faculty archive).

Clinically, TAO most often presents in men younger than 50 years with distal ischemia, ischemic ulcers, or gangrene [1]. Owing to the absence of pathognomonic clinical, radiological, or histopathological findings, the diagnosis is primarily clinical and based on exclusion criteria, supported by characteristic angiographic features such as corkscrew collaterals [1,11] (Figure 1). Despite extensive investigation, both the pathogenesis and prognosis of TAO remain unclear.

Thromboxane A_2_ (TXA_2_) and prostacyclin (PGI_2_) play important and opposing roles in the pathogenesis of thrombotic events. TXA_2_ promotes platelet activation and induces microvascular vasoconstriction, thereby facilitating thrombus formation, whereas PGI_2_, the physiological antagonist of TXA_2_, exerts vasodilatory effects and inhibits platelet aggregation within the circulation [4]. Consequently, a pathological imbalance favoring TXA_2_ over PGI_2_ may predispose to thrombosis. In this context, iloprost, a synthetic prostacyclin analog, has been shown to act as an effective vasodilatory agent in the treatment of Buerger’s disease [1].

With advances in imaging technology, novel modalities have been introduced for the evaluation of peripheral vascular disease. CT perfusion, initially developed for organ perfusion assessment, has emerged as a quantitative tool for evaluating distal perfusion in PAD. Previous studies have demonstrated improved distal perfusion following angioplasty using CT perfusion imaging [12,13]. Despite certain limitations, including radiation exposure, cost, and availability, the relatively low-dose protocols, operator independence, and anatomical stability of the extremities make CT perfusion a practical and reproducible option in selected patients.

Current clinical guidelines are not addressed in detail in this section, as Buerger’s disease remains a condition without a definitive curative treatment. Owing to the lack of universally accepted management strategies, TAO is generally considered within the spectrum of chronic limb-threatening ischemia. Therapeutic approaches vary considerably between institutions and regions, and standardized, evidence-based guidelines have yet to be established.

At present, smoking cessation remains the only universally accepted cornerstone of treatment [3,4]. Pharmacological therapies provide limited benefit; however, iloprost has been shown to improve symptoms and reduce the risk of amputation [14], while oral agents demonstrate modest efficacy [14,15]. In refractory cases, sympathectomy or endovascular interventions may be considered, although both surgical and endovascular approaches remain controversial and are frequently avoided due to technical challenges and variable outcomes [15,16,17].

There is currently no universally accepted invasive treatment protocol for TAO [18]. Among available options, endovascular intervention may be preferred in cases of limb-threatening occlusions to restore perfusion and facilitate early limb salvage [19]. Surgical revascularization is generally not considered a first-line approach because of limited distal targets, graft availability, and access challenges. Nevertheless, in carefully selected patients with suitable distal arteries and active wounds, bypass surgery may contribute to limb preservation [20,21]. Factors such as vasospasm, poor distal runoff, disease progression, and myointimal hyperplasia significantly limit long-term bypass patency [20]. Overall, both endovascular and surgical strategies aim to improve distal perfusion and promote wound healing in order to prevent amputation, although long-term outcomes remain suboptimal [2,3].

### Aim

The primary aim of this study was to evaluate the therapeutic efficacy of intravenous iloprost in patients with thromboangiitis obliterans (Buerger’s disease) using a combination of clinical, hemodynamic, and quantitative imaging parameters. Specifically, the study assessed changes in ankle–brachial index, pain-free walking distance, and lower-extremity perfusion and functional capacity as measured by CT perfusion imaging.

The secondary aim was to investigate the feasibility of CT perfusion as a reproducible and objective imaging modality for monitoring microvascular perfusion dynamics and treatment response in patients with TAO.

Overall, this study sought to clarify the impact of iloprost treatment duration on clinical and quantitatively measurable outcomes and to contribute to the development of more precise and objective monitoring strategies in the management of Buerger’s disease.

## 2. Materials and Methods

### 2.1. Patient Population

This study was initiated following the approval of the Clinical Research Ethics Committee of Istanbul University, Istanbul Faculty of Medicine (Decision No. 1924856, dated 19 July 2023). Informed consent was obtained from all patients or their legal guardians after providing detailed information about the study’s objectives, procedures, data use, and follow-up assessments. Previously hospitalized patients were included using their signed consent forms. Participation was voluntary, and patients were informed of their right to withdraw at any time without consequence.

Patients with a definitive diagnosis of Buerger’s disease (thromboangiitis obliterans) were included based on established clinical and angiographic criteria. Clinical features comprised a history of tobacco use, disease onset before 50 years of age, distal extremity ischemia (rest pain, ischemic ulcers, or gangrene), and the absence of major atherosclerotic risk factors other than smoking. Imaging evaluation demonstrated characteristic angiographic findings, including segmental distal arterial occlusions with corkscrew collateral vessels, without evidence of proximal atherosclerotic disease. All patients were under regular follow-up at the Department of Cardiovascular Surgery and fulfilled accepted diagnostic criteria for TAO (Shionoya criteria) [22]. Eligible patients had received intravenous iloprost therapy and underwent CT perfusion imaging both before and after treatment. Only those with complete clinical data and consistent follow-up records were selected. The study included 33 patients (32 men and 1 woman) aged between 31 and 57 years.

### 2.2. Methodology

Retrospective data were collected from routine outpatient records, including complaints, walking distance, smoking status, medication use, ABI, lifestyle and activity level, lower limb perfusion assessments, noninvasive and invasive vascular imaging, as well as hemogram, biochemical, and coagulation profiles.

Post-treatment follow-up evaluations were performed during routine clinical visits. ABI and walking distance measurements were conducted by physicians from the same department, while follow-up examinations were carried out by clinicians blinded to the study protocol. The presence of rest pain, ischemic ulcers, amputations, or previous surgical/interventional procedures was not a criterion for exclusion.

Walking distance was measured at each visit on a LifeGear^®^ treadmill (LifeGear Inc., Gardena, CA, USA; manufactured in Shanghai, China) (3 km/h, 10% incline); claudication was defined as the distance at which calf pain occurred, with ≥1500 m considered pain-free.

### 2.3. Intravenous Iloprost Protocol

Iloprost was administered daily via intravenous infusion diluted in 5% dextrose, with dose titration from 0.5 ng/kg/min to a maximum of 2.0 ng/kg/min based on patient tolerance. Each infusion lasted six hours and was delivered through a forearm vein. Side effects guided dose adjustments.

At follow-up, pain-free walking distance (treadmill), ABI, smoking status, and medication adherence were assessed. Due to the objectivity of the records, pain scores were not included in the evaluation.

Patients were grouped by treatment duration: group 1: no IVI (n = 8), group 2: 7 days (n = 7), group 3: 14 days (n = 10), group 4: 21 days (n = 8), totaling 33 patients (Table 1).

### 2.4. CT Perfusion Protocol and Analysis

All perfusion CT scans were performed using a 640-slice scanner (Aquilion ONE^®^, Canon Medical Systems, Tokyo, Japan). To minimize movement, patients’ legs were positioned with padded stabilizers. A bolus of 60 mL iodinated contrast (Omnipaque^®^ 350, GE Healthcare, Oslo, Norway) was injected via a superficial upper extremity vein at 5 mL/s, followed by 20 mL saline flush. Volumetric acquisition (16 cm Z-axis coverage) was performed without table movement, covering the distal tibia bilaterally. Scanning parameters: 120 kVp, 120 mAs, 0.5 s rotation time, with multiphase acquisitions at 3-s intervals. Radiation dose per exam was 4–6 mSv.

**Figure 2:**
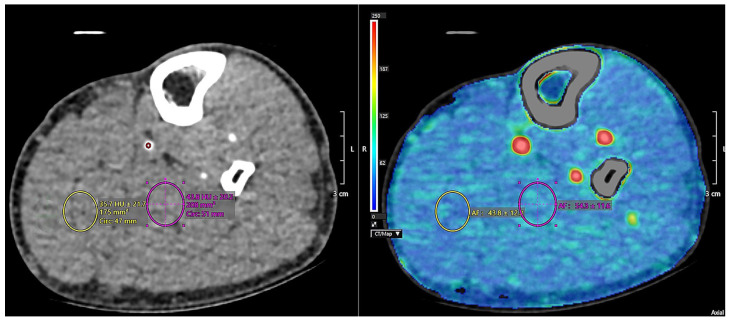
Perfusion computed tomography section.

**Figure 3:**
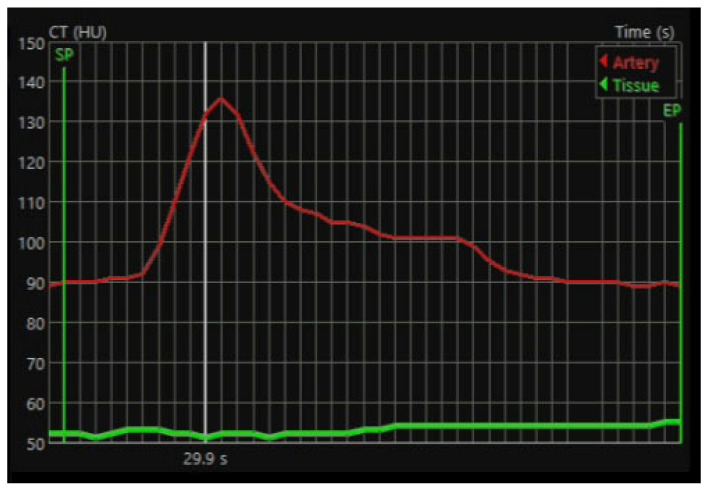
Arterial flow and tissue perfusion measurement graph at the workstation. White line: The moment of measurement of arterial flow and tissue perfusion (29.9 sec).

All images were transferred in DICOM format and analyzed using Vitrea™FX version 7.14, vital images, Inc., Minnetoka, MN, USA Two experienced radiologists independently reviewed the series, blinded and randomized to pre- and post-iloprost status. For each extremity, a region of interest (ROI) was manually placed on the dominant artery at the phase demonstrating maximal arterial enhancement. The ROI was propagated across all phases and adjusted when necessary. Additional ROIs were then placed within muscle tissue regions devoid of visible vessels and minimal fatty infiltration to assess tissue perfusion. Arterial flow (AF) values were calculated, and measurements were repeated three times for each extremity, with mean values recorded. All analyses were performed on the symptomatic limb for each patient and independently repeated by both radiologists (Figure 2 and Figure 3).

### 2.5. Statistical Analysis

Continuous variables are presented as mean ± standard deviation, median, and minimum–maximum values, as appropriate. Categorical variables are expressed as frequencies and percentages. Comparisons of categorical data were performed using the chi-square test or Fisher’s exact test, as appropriate. Pre- and post-treatment comparisons were analyzed using the Wilcoxon signed-rank test for the overall study population as well as for subgroups defined according to treatment duration. Differences among the four treatment groups were assessed using the Kruskal–Wallis test, and intergroup comparisons were further evaluated using the Kruskal–Wallis Z test. A two-sided *p* value < 0.05 was considered statistically significant. All statistical analyses were performed using SPSS software, version 29.0 (IBM Corp., Armonk, NY, USA).

In TAO, the pathology of small- and medium-sized arteries reduces distal flow and perfusion reserve. Intravenous iloprost, a prostaglandin analog proven beneficial in TAO. We aimed to evaluate its quantitative effect on microvascular and distal tissue perfusion using CT perfusion imaging.

No artificial intelligence–based tools or generative artificial intelligence technologies were used in the design, analysis, or preparation of this manuscript. A translation program was used solely for language editing and grammatical correction purposes, without any influence on the scientific content.

## 3. Results

In one patient in the study, data on claudication after iloprost was missing; in another, activation phase records were missing; apart from that, no data loss occurred. The cohort included 33 patients (32 men and 1 woman), aged 31–57 (mean: 42.9). Mean disease duration was 8.1 years. Active smoking was present in 51.5%. At admission, 51.5% had active symptoms, and 33.3% had open ulcers. Minor amputations were observed in 3 patients, and major amputations in 2. Comorbidities were present in 36.4%; 72.7% maintained an active daily life. All of patient received dual antiplatelet therapy; no patient underwent spinal cord stimulation. Angioplasty was reported in 48.5%, surgical revascularization in 9%, and one had a lumbar sympathectomy (Table 2). Only one patient was excluded from the study due to severe leg edema and infection. No serious side effects requiring discontinuation of iloprost treatment were reported.

Arterial flow measurements from CT perfusion were compared with ABI and claudication distance, with 32 patients (97%) included in the analysis; one patient (3%) was excluded due to severe leg edema and skin infection.

Ankle–brachial index values before and after treatment were compared within each group; no statistically significant difference was observed before and after intravenous iloprost treatment in Group 1 and Group 2, while a statistically significant increase was observed in Group 3 and Group 4 (Table 3).

When claudication distances before and after iloprost treatment were calculated and compared within each group, no statistically significant change was observed in Group 1. Although Groups 2 and 3 showed a positive trend according to the Wilcoxon Signed Ranks Test, the results were not statistically significant. In Group 4, a statistically significant increase in claudication distance was detected after iloprost treatment (Table 4).

When CT perfusion arterial flow measurements before and after iloprost treatment were calculated and compared within each group, Groups 1 and 2 showed a positive trend according to the Wilcoxon Signed Ranks Test, but the results were not statistically significant. In Groups 3 and 4, a statistically significant increase in tissue perfusion was observed after iloprost treatment (Table 5).

When the treatment groups were compared, no statistically significant difference was observed between the untreated group and the 7-day treatment group. Similarly, no significant difference was found between the 14-day and 7-day groups. Although there was no significant difference in ABI or claudication distance between the 14-day and untreated groups, CT perfusion measurements showed a statistically significant difference (*p* = 0.001). The 21-day group demonstrated statistically significant improvements in ABI, claudication distance, and CT perfusion compared to the untreated group. When comparing the 21-day and 7-day groups, ABI and claudication distance showed significant differences, while CT perfusion values did not (Table 6). In this comparison, a statistically significant positive effect of iloprost treatment was observed within both the 14-day and 21-day groups compared to the other groups. No significant differences were observed between the 14-day and 21-day groups across any of the three parameters. This suggests that extending IV iloprost treatment from 14 to 21 days may not provide additional benefit.

According to the Wilcoxon Signed Rank test results, the table shows that iloprost treatment generally has a positive effect on improving ABI and CT perfusion values in the early period but does not provide a significant improvement in claudication distance.

The ankle–brachial index, routinely assessed at every clinical visit as an indicator of disease status, was evaluated for its usefulness in monitoring limb perfusion. Patients with an ABI value of 0.8 or higher who received intravenous iloprost therapy demonstrated a statistically significantly greater increase in arterial flow on CT perfusion measurements. In contrast, patients with ABI values below 0.8 exhibited a comparatively smaller change in limb perfusion. These findings suggest that an ABI threshold of 0.8 may represent a critical cutoff for achieving optimal therapeutic benefit from iloprost treatment (Table 7).

## 4. Discussion

Non-invasive methods to improve limb blood flow include medical therapies aimed at enhancing collateral circulation, reducing platelet adhesion and aggregation, suppressing inflammatory mediators, improving endothelial function, promoting vasodilation, and increasing erythrocyte flexibility.

Intravenous iloprost is a medical treatment shown to be effective in TAO, though no consensus exists on optimal duration [23]. It is particularly useful for patients in the active phase who lack suitable distal vessels for bypass or access for endovascular intervention [24]. In a randomized, double-blind trial, TAO patients with rest pain received either IV iloprost or low-dose aspirin for 28 days. Both groups improved, with better outcomes in the iloprost group at 6 months, especially in pain relief and ulcer healing [23].

Previous studies suggest that a 28-day regimen may offer greater benefit than shorter durations [25]. In our study, patients receiving 7, 14, or 21 days of iloprost were compared to untreated controls using ABI, claudication distance, and CT perfusion-derived arterial flow values. Due to incomplete and subjective pain data, the effect of iloprost on pain was not evaluated.

The ankle–brachial index is a non-invasive, low-cost, and reliable method for assessing limb perfusion. CT perfusion in PAD—A study examining the correlation with angiographic and hemodynamic parameters demonstrated the applicability of perfusion CT in the evaluation of PAD [12,13]. In our study, ABI, claudication distance, and CT perfusion measurements were compared between untreated patients and those who received IV iloprost for 7, 14, or 21 days to assess treatment efficacy. CT perfusion–derived arterial flow values enabled quantitative evaluation of therapeutic response.

Our results showed no significant change in ABI was found in the untreated and 7-day iloprost groups, while significant increases were observed in the 14- and 21-day groups. Claudication distance did not change significantly in the untreated, 7-day, or 14-day groups, though a positive trend was noted; only the 21-day group showed a significant increase. CT perfusion values improved in all iloprost groups, but significance was reached only in the 14- and 21-day groups, suggesting measurable perfusion benefits after two weeks of treatment.

No significant difference was found between the untreated and 7-day groups, suggesting that 7 days may be insufficient for treatment. While no difference was found in ABI and CI between the 14-day and untreated groups, a significant difference was found in CT perfusion. This result demonstrates that CT perfusion scores improve early after effective treatment compared to clinical findings.

A significant difference was observed between the 21-day iloprost group and the untreated group in three parameters (ABI, claudication distance, and arterial flow). We believe that this significant result may be related to the dosage or duration of iloprost administration.

No significant difference was observed between the 21-day and 14-day groups in any parameter, suggesting that 14-day iloprost treatment may be as effective and sufficient as 21-day treatment. From a clinical and health policy perspective, this observation may be relevant, particularly considering treatment costs and resource utilization. While clinical improvement was more evident in the 21-day group, the absence of statistically significant differences between the two groups, together with the early improvement in CT perfusion at day 14, underscores the potential value of CT perfusion imaging for early treatment assessment rather than definitive clinical decision-making.

In this study, when ABI values were examined across ranges, no significant difference in perfusion was found with iloprost treatment in patients with an ABI > 1 or >0.9, while a significantly greater increase in CT perfusion was observed after treatment in patients with an ABI > 0.8. This finding should be interpreted cautiously. Given the distal, segmental nature of TAO and the presence of extensive collateral circulation, ABI may not fully capture microvascular perfusion status. The observed perfusion response in patients with moderately preserved ABI values may suggest that earlier intervention, prior to advanced tissue damage, allows a more measurable perfusion response, a concept that warrants further investigation in prospective studies.

Overall, the findings of this study support the feasibility of CT perfusion as a complementary imaging tool for detecting early changes in limb perfusion following iloprost therapy. However, given the retrospective design, small subgroup sizes, and lack of validated clinical endpoints, the results should be interpreted with caution and primarily viewed as hypothesis-generating. Larger, prospective, and ideally randomized studies incorporating robust clinical outcomes are needed to define the optimal role of CT perfusion imaging and to clarify the most effective treatment duration of intravenous iloprost in TAO.

### Limitations

This study has several limitations. First, its retrospective design inherently limits causal inference. Within the analyzed cohort, patients who received 7 days of treatment appeared to have milder symptoms and a better baseline clinical condition compared with those treated for longer durations, which may have influenced the lack of statistically significant differences observed in some outcome measures.

Second, the study population exhibited heterogeneous clinical characteristics, which may have contributed to variability in disease presentation and post-treatment responses. In addition, the evaluated outcomes reflect early responses recorded during hospitalization rather than long-term clinical effects.

Although improvements in CT perfusion were observed in the 14- and 21-day iloprost groups compared with untreated patients, the durability and clinical relevance of these findings remain uncertain. As the maximum treatment duration in this study was limited to 21 days, the potential benefits of extended iloprost therapy could not be assessed. Further prospective studies with longer follow-up are therefore needed to better define long-term outcomes.

## 5. Conclusions

This study demonstrates that intravenous iloprost therapy leads to measurable improvements in limb perfusion in patients with thromboangiitis obliterans, with treatment duration playing a critical role in therapeutic response. While a short-term treatment of 7 days did not result in significant clinical or perfusion-related improvements, longer treatment durations were associated with progressively greater benefits. In particular, CT perfusion imaging revealed significant improvements as early as day 14, preceding detectable changes in conventional clinical parameters such as ankle–brachial index and claudication distance.

The findings further indicate that a 14-day course of iloprost may provide perfusion benefits comparable to those observed with 21-day treatment, suggesting that extended treatment beyond two weeks may not yield additional short-term perfusion advantages. From a clinical and health-economic perspective, this supports the potential adequacy of a 14-day iloprost regimen in selected patients. Moreover, the observed dissociation between early perfusion improvement and delayed clinical response highlights the value of CT perfusion as a sensitive and objective tool for early treatment monitoring.

In addition, subgroup analysis based on ABI values suggests that patients with moderate disease severity, particularly those with an ABI ≥ 0.8, derive greater perfusion benefit from iloprost therapy. This finding underscores the importance of timely intervention before advanced disease progression.

Overall, this study supports the role of CT perfusion imaging as a quantitative modality for assessing treatment response and optimizing therapy duration in Buerger’s disease. Larger prospective studies are warranted to validate these findings and to further define individualized treatment strategies aimed at improving clinical outcomes and limb salvage in this challenging patient population.

## Figures and Tables

**Table 1:** Number of patients and treatment durations in the working groups.

	Group 1	Group 2	Group 3	Group 4
n (number of patients)	8	7	10	8
Treatment (day)	0	7	14	21

**Table 2:** Clinical characteristics of patients.

	Yes (n)	No %	Yes (n)	No %
Smoking	17	51.5%	16	48.5%
Active complaint	17	51.5%	16	45.5%
Active peripheral ulcer	11	33.3%	22	66.7%
Upper extremity involvement	3	9%	30	91%
Organ involvement	1	3%	32	97%
Migratory thrombophlebitis	11	33.3%	22	66.7%
Active lifestyle	24	72.7%	9	27.3%
Additional disease Diabetes Hypertension	12	36.4% 24% 12.4%	21	63.6%

**Table 3:** Ankle brachial index values of the groups before and after iloprost.

		n	Min–Max	Mean ± sd.	Median	*p* Value
Group 1	ABI ^a^		0.38–1.26	0.89 ± 0.2	0.90	Z = −1.4
	ABI ^b^	8	0.44–1.27	0.90 ± 0.2	0.91	*p* = 0.14
Group 2	ABI ^a^		0.41–1.15	0.68 ± 0.3	0.55	Z = −1
	ABI ^b^	7	0.44–1.24	0.74 ± 0.3	0.59	*p* = 0.34
Group 3	ABI ^a^		0.00–0.93	0.54 ± 0.3	0.62	Z = −2.5
	ABI ^b^	9	0.00–1.04	0.62 ± 0.3	0.72	*p* = 0.012 *
Group 4	ABI ^a^		0.37–1.00	0.71 ± 0.2	0.75	Z = −2.5
	ABI ^b^	8	0.50–1.16	0.90 ± 0.2	0.97	*p* = 0.012 *

^a^: before iloprost, ^b^: after iloprost, ABI: ankle–brachial index, bold and *: *p* < 0.05. Wilcoxon Signed Ranks Test.

**Table 4:** Claudication values of the groups before and after iloprost.

		n	Min–Max (m)	Mean ± sd. (m)	Median (m)	*p* Value
Group 1	CI ^a^	8	30–5000	3766 ± 2284	5000	Z = 0
	CI ^b^		30–5000	3766 ± 2284	5000	*p* = 0.99
Group 2	CI ^a^	7	50–5000	2914 ± 2602	5000	Z = −1
	CI ^b^		50–5000	2918 ± 2597	5000	*p* = 0.31
Group 3	CI ^a^	9	30–5000	2394 ± 2487	1000	Z = −1.6
	CI ^b^		10–5000	2450 ± 2431	1000	*p* = 0.1
Group 4	CI ^a^	8	20–5000	790 ± 1707	215	Z = −2.3
	CI ^b^		50–5000	912 ±1665	425	*p* = 0.01 *

^a^: before iloprost, ^b^: after iloprost, CI: claudication intermittent, bold and *: *p* < 0.05. Wilcoxon Signed Ranks Test.

**Table 5:** Arterial flow values of the groups before and after iloprost.

		n	Min–Max (AF)	Mean ± sd. (AF)	Median (AF)	*p* Value
Group 1	CT ^a^		27.5–81.3	51.06 ± 19.0	44.3	Z = 0
	CT ^b^	8	29.0–82.4	52.27 ± 18.5	46.7	*p* = 0.32
Group 2	CT ^a^		31.5–50.0	40.94 ± 6.6	43.5	Z = −1.5
	CT ^b^	7	34.3–89.2	51.48 ± 19.1	44.6	*p* = 0.128
Group 3	CT ^a^		19.2–68.5	36.57 ± 15.8	30.7	Z = −2.6
	CT ^b^	9	35.2–89.9	52.92 ± 20.1	44.5	*p* = 0.008 *
Group 4	CT ^a^		25.0–63.0	40.38 ± 11.9	40.8	Z = −2.5
	CT ^b^	8	50.0–102.0	70.92 ± 17.4	69.9	*p* = 0.012 *

^a^: before iloprost, ^b^: after iloprost, CT: computed tomography, AF: arterial flow, bold and *: *p* < 0.05. Wilcoxon Signed Ranks Test.

**Table 6:** Statistical comparison between the groups.

	*p* Value		
	ABI	CI	CT Perfusion (AF)
Group 2–Group 1	0.9	0.99	0.99
Group 3–Group 1	0.35	0.82	0.001 *
Group 3–Group 2	0.99	0.99	0.78
Group 4–Group 1	0.0001 *	0.002 *	0.001 *
Group 4–Group 2	0.006 *	0.016 *	0.43
Group 4–Group 3	0.08	0.18	0.51

ABI: ankle brachial index, CI: claudication intermittent, CT: computed tomography, AF: arterial flow, bold and *: *p* < 0.05. Pairwise comparisons.

**Table 7:** Statistical evaluation of patients with ABI values > 0.8.

	CT ^b^ − CT ^a^ Perf. Difference
Mann–Whitney U	61
Wilcoxon W	139
Z	−2.29
***p*** (2-tailed)	0.02 *

^a^: before iloprost, ^b^: after iloprost, CT: computed tomography, bold and *: *p* < 0.05.

## Data Availability

The data supporting the findings of this study are available from the corresponding author upon reasonable request.

## Symbols and Abbreviations

AF	Arterial Flow
ABI	Ankle–Brachial Index
ANCA	Antineutrophil Cytoplasmic Antibody
ASO	Atherosclerosis Obliterans
CT	Computed Tomography
cAMP	Cyclic Adenosine Monophosphate
CI	Intermittent Claudication
CRP	C-Reactive Protein
DICOM	Digital Imaging and Communications in Medicine
DM	Diabetes Mellitus
DMAH	Low-Molecular-Weight Heparin
DSA	Digital Subtraction Angiography
HBOT	Hyperbaric Oxygen Therapy
HL	Hyperlipidemia
HLA	Human Leukocyte Antigen
HT	Hypertension
ICAM	Intercellular Adhesion Molecule
Ig	Immunoglobulin
IVI	Intravenous Iloprost
kg	Kilogram
CHF	Congestive Heart Failure
m	Meter
Max	Maximum
Min	Minimum
mL	Milliliter
SCS	Spinal Cord Stimulator
n	Number of patients

## References

[1] Mills J.L. (2003). Buerger’s disease in the 21st century: Diagnosis, clinical features, and therapy. Semin. Vasc. Surg..

[2] Watanabe Y., Shimizu Y., Hashimoto T., Iwahashi T., Shigematsu K., Nakaoka Y., Harigai M., Japan Research Committee of the Ministry of Health, Labour, and Welfare for Intractable Vasculitis (JPVAS) (2024). Demographic Traits, Clinical Status, and Comorbidities of Patients With Thromboangiitis Obliterans in Japan. Circ. J..

[3] Zheng P., Wang W. (2024). Etiology and Pathogenesis of Buerger’s Disease. Probing Selected Autoimmune Diseases for Focused Perspectives.

[4] Shapouri-Moghaddam A., Saeed Modaghegh M.H., Rahimi H.R., Ehteshamfar S.M., Tavakol Afshari J. (2019). Molecular mechanisms regulating immune responses in thromboangiitis obliterans: A comprehensive review. Iran. J. Basic Med. Sci..

[5] Makita S., Nakamura M., Murakami H., Komoda K., Kawazoe K., Hiramori K. (1996). Impaired endothelium-dependent vasorelaxation in peripheral vasculature of patients with thromboangiitis obliterans (Buerger’s disease). Circulation.

[6] Małecki R., Zdrojowy K., Adamiec R. (2009). Thromboangiitis obliterans in the 21st century—A new face of disease. Atherosclerosis.

[7] Eichhorn J., Sima D., Lindschau C., Turowski A., Schmidt H., Schneider W., Haller H., Luft F.C. (1998). Antiendothelial cell antibodies in thromboangiitis obliterans. Am. J. Med. Sci..

[8] Kobayashi M., Ito M., Nakagawa A., Nishikimi N., Nimura Y. (1999). Immunohistochemical analysis of arterial wall cellular infiltration in Buerger’s disease (endarteritis obliterans). J. Vasc. Surg..

[9] Slavov E.S., Stanilova S.A., Petkov D.P., Dobreva Z.G. (2005). Cytokine production in thromboangiitis obliterans patients: New evidence for an immune-mediated inflammatory disorder. Clin. Exp. Rheumatol..

[10] Fazeli B., Ravari H., Farzadnia M. (2012). Does a species of Rickettsia play a role in the pathophysiology of Buerger’s disease?. Vascular.

[11] Suzuki S., Mine H., Umehara I., Yoshida T., Okada Y. (1982). Buerger’s disease (thromboangiitis obliterans): An analysis of the arteriograms of 119 cases. Clin. Radiol..

[12] Cacione D.G., Macedo C.R., do Carmo Novaes F., Baptista-Silva J.C. (2020). Pharmacological treatment for Buerger’s disease. Cochrane Database Syst. Rev..

[13] Gao J., Huang L., Wang J. (2021). Outcomes of Anticoagulant Therapy with Low-Molecular-Weight Heparin (LMWH) and Warfarin for Thromboangiitis Obliterans (TAO). Curr. Vasc. Pharmacol..

[14] Ghoneim B.M., Karmota A.G., Abuhadema A.M., Shaker A.A., Abdelmawla H.M., Nasser M.M., Elmahdy H.Y., Mostafa H.A., Khairy H.M. (2019). Management of Buerger’s Disease in Endovascular Era. Int. J. Angiol..

[15] Kim D.H., Ko Y.G., Ahn C.M., Shin D.H., Kim J.S., Kim B.K., Choi D., Hong M.-K., Jang Y. (2018). Immediate and late outcomes of endovascular therapy for lower extremity arteries in Buerger disease. J. Vasc. Surg..

[16] Isobe M., Amano K., Arimura Y., Ishizu A., Ito S., Kaname S., Kobayashi S., Komagata Y., Komuro I., Komori K. (2020). JCS2017 Guideline on Management of Vasculitis Syndrome—Digest Version—. Circ. J..

[17] Sah B.R., Veit-Haibach P., Strobel K., Banyai M., Huellner M.W. (2019). CT-perfusion in peripheral arterial disease—Correlation with angiographic and hemodynamic parameters. PLoS ONE.

[18] Cindil E., Erbas G., Akkan K., Cerit M.N., Sendur H.N., Zor M.H., Ilgıt E. (2020). Dynamic Volume Perfusion CT of the Foot in Critical Limb Ischemia: Response to Percutaneous Revascularization. Am. J. Roentgenol..

[19] Graziani L., Morelli L., Parini F., Franceschini L., Spano P., Calza S., Sigala S. (2012). Clinical outcome after extended endovascular recanalisation in Buerger’s disease in 20 consecutive cases. Ann. Vasc. Surg..

[20] Sasajima T., Kubo Y., Inaba M., Goh K., Azuma N. (1997). Role of infra inguinal bypass in Buerger’s disease: An eighteen-year experience. Eur. J. Vasc. Endovasc. Surg..

[21] De Caridi G., Massara M., Villari S., Martelli E., Spinelli F., Grande R., Butrico L., de Franciscis S., Serra R. (2016). Extreme distal bypass to improve wound healing in Buerger’s disease. Int. Wound J..

[22] Shionoya S. (1998). Diagnostic criteria of Buerger’s disease. Int. J. Cardiol..

[23] Fiessinger J.N., Schäfer M. (1990). Trial of iloprost versus aspirin treatment for critical limb ischaemia of thromboangiitis obliterans. Lancet.

[24] Chen Q., Chen J., Li J., Cheng Y., Zhang R., Liu Z. (2023). Recent advances of oxidative stress in thromboangiitis obliterans: Biomolecular mechanisms, biomarkers, sources and clinical applications. Thromb. Res..

[25] Norgren L., Alwmark A., Angqvist K.A., Hedberg B., Bergqvist D., Takolander R., Claes G., Lundell A., Holm J., Jivegärd L. (1990). A stable prostacyclin analogue (iloprost) in the treatment of ischaemic ulcers of the lower limb: A Scandinavian-Polish placebo controlled, randomized multicenter study. Eur. J. Vasc. Surg..

